# Weathering of a Polyurethane-Based Gel Electrolyte

**DOI:** 10.3390/polym15061448

**Published:** 2023-03-14

**Authors:** Christopher Johannes, Michael Hartung, Hans-Peter Heim

**Affiliations:** Institute of Material Engineering, Plastics Engineering, University of Kassel, 34125 Kassel, Germany

**Keywords:** weathering, polyurethane electrolyte, flexible electrochromic device

## Abstract

A recently described flexible polyurethane electrolyte was artificially weathered at 25/50 °C and 50% r.h. in air and at 25 °C in a dry nitrogen atmosphere, each with and without UV irradiation. Different formulations and the polymer matrix, used as a reference, were weathered in order to investigate the influence of the amount of conductive lithium salt and the solvent propylene carbonate. The complete loss of the solvent at a standard climate was already observed after a few days, strongly influencing the conductivity and mechanical properties. The essential degradation mechanism appears to be the photo-oxidative degradation of the polyol’s ether bonds, which leads to chain scission, oxidation products and negative changes in the mechanical and optical properties. A higher salt content has no effect on the degradation; however, the presence of propylene carbonate intensifies the degradation.

## 1. Introduction

Polyurethane, as a matrix in polymer electrolytes, has been studied in particular because of its hard–soft structure, which contains hard isocyanate segments and soft polyol segments [1,2,3,4,5]. Recently, a new cold-curing, polyurethane-based gel electrolyte was characterized and its application in an electrochromic (EC) multilayer film was demonstrated [6]. In addition to its high light transmittance and colorlessness, the electrolyte’s mechanical strength and flexibility allow for the deformation of EC films. There is a growing demand for flexible electrolytes in stretchable or deformable systems, such as sensors or foldable displays [7,8]. Using plastic back-injection molding of flexible EC films, the author intends to fabricate three-dimensionally shaped dimmable window panes for demonstration purposes (for example, in automobiles). Due to the effects of environmental influences such as temperature, UV radiation and oxygen during outdoor applications, aging phenomena are to be expected. As the root causes of aging can be manifold in a system such as an EC device, it is useful to first examine the device components individually. In order to understand the electrolyte’s basic aging mechanisms, it was weathered and characterized with respect to its ionic conductivity and optical, mechanical and chemical properties after defined exposure durations in this study.

The electrolyte is based on an elastomeric polyurethane (PUR) matrix to which lithium salt and solvent were added. The PUR matrix is a cold-curing casting system and is prepared from an aliphatic diisocyanate prepolymer, synthesized by HDI, and a trifunctional polyether polyol. The trifunctional polyol leads to a wide-mesh, crosslinked matrix. The resistance of PUR to weathering effects such as temperature, humidity and global radiation has been widely researched [9,10], as it is a widespread material in industrial and consumer products. However, because it is an entire class of materials, differentiation must be performed with respect to its specific structural makeup. Additionally, the salt and solvent additives could have an influence on the aging behavior.

PUR is formed by a polyaddition reaction of polyisocyanates with polyols. A distinction is essentially made between polyether and polyester polyols on one hand and aromatic and aliphatic polyisocyanates on the other. The polyether polyols play a predominant role economically. Among the polyisocyanates, aromatics play a more important role than aliphatics because of their greater reactivity and economy. Aliphatic polyisocyanates, however, are used in products such as paints or coatings due to their better lightfastness. The basic reaction is the reaction of an isocyanate group (NCO group) with a hydroxyl group (OH group) to form the urethane group. In addition to this basic reaction, there are other possible reactants of isocyanate groups with other than OH groups, particularly in case of an NCO surplus. These are, above all, NH_2_ groups of amines which react to form urea and water, which then reacts to form foamed polyurethanes with the elimination of CO_2_. Reactions of isocyanates with each other or of reaction products from these basic reactions are also possible. Since the urethane group is not necessarily formed in these reactions, the more appropriate and broader term isocyanate chemistry is also used. Even if they do occur, the urethane groups usually determine the properties of the material much less than the high-molecular-weight polyol components (i.e., when the ratio of the molar masses of urethane groups to the total mass is very small). [11]

In this context, the resistance of polyurethanes to weathering regularly depends on the polyol components used for synthesis. Those with polyester polyols are considered more resistant than those with polyether polyols. The greater susceptibility is based on the greater tendency of the ether groups to oxidize, especially at elevated temperatures (thermal oxidation), which ultimately leads to chain scission. Peroxides, acids, aldehydes and esters can be formed. For this reason, antioxidants are often used. However, polyether polyurethanes hydrolyze only very slowly. [12] Thermal decomposition without oxygen takes place at over 250 °C in the first stage due to thermolysis of urethane linkages and in the second stage due to decomposition of the polyol component [9]. This fits well with the previous result of a thermogravimetric analysis of the subjected polyurethane-based electrolyte [6]. Sui et al. reported that during prolonged temperature storage, degradation of the hard (isocyanate dominated) segment took place first at approximately 175 °C through scission of the urethane bond to form urea, alcohols and CO_2_ [13]. According to Ehrenstein et al., the urethane grouping is stable at service temperatures of up to 80 °C [10]. Cold hardening, i.e., an increase in hardness and a decrease in elasticity, can be observed at room temperature and under low, continuous stress in hot casting systems. This is attributed to increasing crystallization in the soft segment. [14] However, this phenomenon is not likely to occur, or to occur only to a lesser extent, in crosslinking systems because a chemically bonded network is formed in those systems that inhibits physical bonding.

The photodegradation mechanism in an aliphatic PUR is comparable to other polymers, such as polyamide. Light with a wavelength of 254 nm (artificially generated or extraterrestrial) leads to the cleavage of the urethane bond. Light with >300 nm that penetrates the Earth’s atmosphere, is not capable of such cleavage but causes the scission of chemical bonds, primarily at the tertiary hydrogen atoms of the main chain but also of the main chain itself (Norrish reactions), leading to free radicals. Through intermediate reactions including oxygen, these radicals lead to the formation of hydroperoxides, which play a decisive role in the degradation of polymer molecules. They decompose by direct absorption or by energy transfer from activated carbonyl or aromatic hydrocarbon groups to form radicals again (photolysis), which in turn leads to further scissions and oxidation products due to their reactivity. This is referred to as a radical-initiated chain reaction. This process not only includes scission but also the crosslinking of polymer chains. For this reason, mechanical properties can develop differently depending on the dominant processes. Nevertheless, aliphatic polyisocyanates, especially those synthesized by hexamethylene diisocyanate (HDI), are considered very stable. However, even if no light-absorbing, so-called chromophoric groups are present in the molecules, chromophores may be present, either due to manufacturing and processing methods (e.g., when using catalysts or due to impurities such as special forms of titanium dioxide) or due to previous thermal oxidation, which occurs in all thermoplastics. [9,10]

Recent publications also proposed decomposition mechanisms of secondary and the tertiary hydroperoxides which also proceed with the formation of water. Furthermore, the urethane bond can be scissioned by back-splitting or hydrolysis, which can be significantly enhanced in the presence of catalysts. However, in the case of elastomers and coatings, this only takes place at temperatures above 200 °C. [10]

Studying an aromatic polyurethane, Abu-Zeid et al. reported that degradation can already be initiated at a temperature of 40 °C and a low dosage UV radiation [15].

Acids or solutions of inorganic salts are thought to have little effect on hydrolysis resistance, but high concentrations and high temperatures can greatly affect the hydrolysis rate. The resistance of PUR cast elastomers to organic hydrocarbons such as oil and gasoline is considered good, but alcohols, organic acids, ketones, and esters are known for their swelling and degrading effects. [16]

Taking into account the above literature, the following hypotheses are made:

- Urethane linkages of the aliphatic PUR are stable because the energy of the (terrestrial) wavelengths is not sufficent for scission reaction;
- Hydrolytic degradation does not occur since polyether polyols are stable and the urethane linkages only hydrolize at very high temperatures;
- Since the aliphatic prepolymer is considered to be particularly lightfast and non-absorbent for UV light, the material does not yellow;
- UV radiation leads to photooxidative degradation within the radical chain reaction of the ether linkages of the polyether polyol, including chain scission, and thus to changes in the structural makeup and properties;
- Weathering in an inert atmosphere slows down the aging mechanisms because there is no/less oxygen for the radical chain reaction;
- Despite the susceptibility of the ether bonds to oxidation, thermo-oxidation does not occur at slightly elevated temperatures (50 °C, without UV radiation);
- The solvent propylene carbonate intensifies the degradation process as it is a carbonate ester, while the amount of salt in the selected experimental space (5/10 wt%) has no effect on the degradation process.

## 2. Materials and Methods

The electrolyte was based on polyurethane (PUR) and prepared in a polyaddition reaction from the trifunctional polyether polyol Desmophen 28HS98 and the diisocyanate Desmodur XP 2617 (both Covestro, Leverkusen, Germany), forming a wide-mesh crosslinked matrix. Lithium trifluoromethanesulfonate (LiTFSi) as a conducting salt and propylene carbonate (both Sigma Aldrich, St. Louis, MO, USA) as a solvent and dibutyl tin dilaurate (DBTL) (Merck KGaA, Darmstadt, Germany) as a curing catalyst were added to the matrix during manufacture. The preparation process and sample types are described in detail in [6], and the electrolyte formulations are listed in Table 1.

Characterization of color properties according to DIN EN ISO 11664-4, using coordinates in the L*a*b* color space CIE 1976, was performed with the spectrophotometer UltraScan Pro from Hunterlab (Reston, VA, USA). Electrochemical impedance spectroscopy was used to determine the bulk resistance, R, of the electrolyte film in a two-electrode arrangement (stainless steel electrodes, 8 mm in diameter, and an initial load of 4.6 N applied by a spring) from rhd instruments (Darmstadt, Germany). The Reference 600 from Gamry Instruments (Warminster, PA, USA) was used as the potentiostat, and a frequency range of 1 MHz to 2 Hz was swept. The specific ionic conductivity, σ, was calculated according to (1)

σ = h/(R × d)(1)

where h is the samples thickness and d represents the area of the electrodes. The tensile test, according to DIN 53504 for elastomers, to determine the tensile strength, R_m_, and the elongation at break *ε*_b_ was performed using the Inspect Table 5 from Hegewald & Peschke Meß- und Prüftechnik GmbH (Nossen, Germany) with a 500 N load cell (200 mm/s at 23 °C, 50% r.h.). For hardness determination (Micro-Shore A) according to DIN ISO 7619-1 for elastomers, the Digitest II from Bareiss Prüfgerätebau GmbH (Oberdischingen, Germany) was used. The measurement was performed on three stacked specimens of 30 mm each. Karl–Fischer titration was used to determine the water content, a DSC Q1000 was used to measure the oxidation induction temperature (OIT), and a TGA Q500 was used to analyze thermal decomposition (both from TA Instruments, New Castle, DE, USA). Fourier-transform infrared spectrometry (FTIR) for chemical analysis was performed using an IRAffinity-1S from Shimadzu (Kyoto, Japan) with an ATR unit (600 to 3800 cm^−1^).

Aging was conducted in a climate chamber, the Atlas SolarClimatic 340 from Weiss Klimatechnik GmbH, equipped with a metal halide lamp, using 1.2 kW to imitate the terrestrial global irradiation (CIE 85). Table 2 shows the different weathering conditions chosen for the experiment. Weathering duration was set to four weeks, with sample extraction and characterization after 3, 7, 14 and 28 days. In order to investigate the aging atmosphere’s influence, one formula was additionally weathered in an inert nitrogen atmosphere in comparison to the oxidizing air atmosphere. For this purpose, a sample container was manufactured consisting of a steel frame with bonded quartz glass (250 × 250 × 2 mm), type FN08, from GVB GmbH (Herzogenrath, Germany) on both sides that had a high transmittance of >90% in the relevant UV spectrum (290–380 nm). Using conventional float glass instead would have filtered a substantial part of the irradiation. The containers cover was not screwable and was equipped with an EPDM gasket. Before weathering, the samples were placed in the container, the cover was screwed tightly and the nitrogen was introduced through a small threaded inlet hole with a small tube. Simultaneously, the concentration of oxygen was measured with an oxygen meter, “Orbmax”, from Orbitalum Tools GmbH (Singen, Germany). As soon as the oxygen concentration fell below 100 ppm (0.01%), the hole was sealed with a screw, which was also equipped with an EPDM gasket. The container was placed in the aging chamber with the glass surface facing towards the irradiation source.

## 3. Results and Discussion

The experimental plan was designed as statistical plan with systematic variation of the influencing factors, salt content (5/10%) and solvent content (10/20%), in order to investigate their influence as well as the interaction effects on the aging behavior of the electrolyte. However, at the selected factor levels for the salt content, there were no statistically significant and relevant effects for any of the investigated properties as hypothesized, nor were there any interaction effects. For this reason, the results of varying the salt content are not described in the text and diagrams for the purpose of clarity.

**Figure 1:**
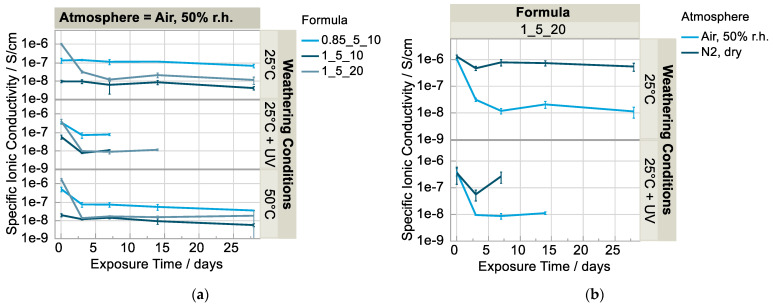
Ionic conductivity over time (n = 3), samples weathered (**a**) in humid air atmosphere (**b**) in dry N_2_ atmosphere vs. humid air.

The electrolyte’s ionic conductivity decreases in any case of the electrolyte’s formula and weathering conditions in the air atmosphere (Figure 1a). The samples (~130 µm thickness), which were exposed to 50 °C and a global irradiation intensity of 50%, could not be measured after three days because they stuck to the container glass and could not be removed without damage. Therefore, they are not shown. Optical changes, an increased gloss and a yellow coloration in particular (Figure 6), also suggested that the material was already severely photodegraded. The same applies to the samples that were exposed to 25 °C and a global irradiation intensity of 50% (notification in figures: 25 °C + UV) for two weeks, with one exception. This shows that an increased temperature accelerates the photodegradation. It is known that a 10 K temperature increase leads to a doubling of the reaction rate. Particularly notable is the strong decrease in ionic conductivity within the first three days of weathering, which is especially evident in the electrolyte formulations with a comparatively high initial ionic conductivity. In this context, the solvent content thus has a negative effect on aging resistance. It is noticeable that, despite weathering, formulation f (0.85_5_10) retains a conductivity at a significantly higher level compared to the other formulations whose values drop comparably to the order of 10^−8^ S/cm. The sub-stoichiometric ratio of the polyurethane matrix of formula f (0.85_5_10) thus seems to have a strong positive effect not only on the conductivity as prepared [6] but also on the aging resistance with respect to conductivity. Interestingly, the samples weathered in the dry nitrogen atmosphere almost retain their conductivity, with a certain variation (Figure 1b). Upon irradiation, however, they seem to degrade similarly, and therefore no measurement was possible after two weeks. The phenomenon that the ionic conductivity of the samples in dry nitrogen (Figure 1b) first decrease (3 days) and then increase approximately to the initial value again (7 days) is not clear.

**Figure 2:**
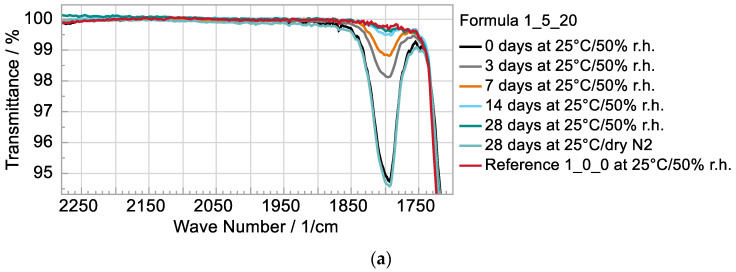

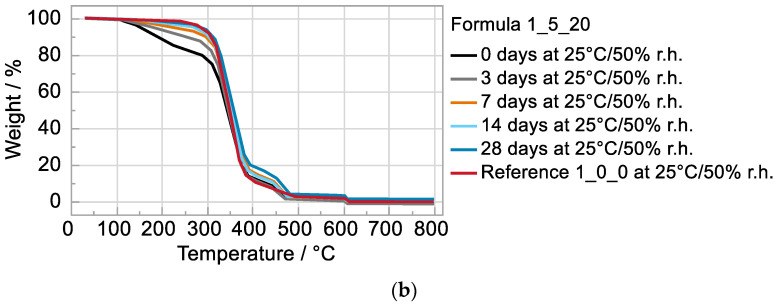
(**a**) FTIR spectra, focused on the solvents peak and (**b**) TGA curves of weathered (25 °C/50% r.h.) samples.

The overlapping curves of the infrared spectroscopy in Figure 2a show for formulation d (1_5_20) with 20 wt% solvent, as an example, that the solvent has already largely escaped after a short time in a moderate climate in air atmosphere (25 °C/50% r.h). The peak at 1800 cm^−1^ can be clearly assigned to the solvent [1]. After 14 days, the curve already approaches the reference sample, the polyurethane matrix without additives, formula a (1_0_0). The same result is seen in the overlapping curves of the TGA measurement (Figure 2b). It is obvious that the loss of the solvent is causal for the strong and fast decrease of the conductivity in the air atmosphere with 50% r.h. Under the dry nitrogen atmosphere, however, the solvent content seems to be unchanged after 28 days (Figure 2a). This explains the retaining conductivity in Figure 1b. A probable explanation is the good solubility of the solvent propylene carbonate in water. Accordingly, it could have migrated into the water in the atmosphere over time. This would be consistent with the faster decrease in conductivity at 50 °C compared to 25 °C because the air contains more water at higher temperatures for the same relative humidity. The water content in the electrolyte is below 2% for all samples at all measuring points.

**Figure 3:**
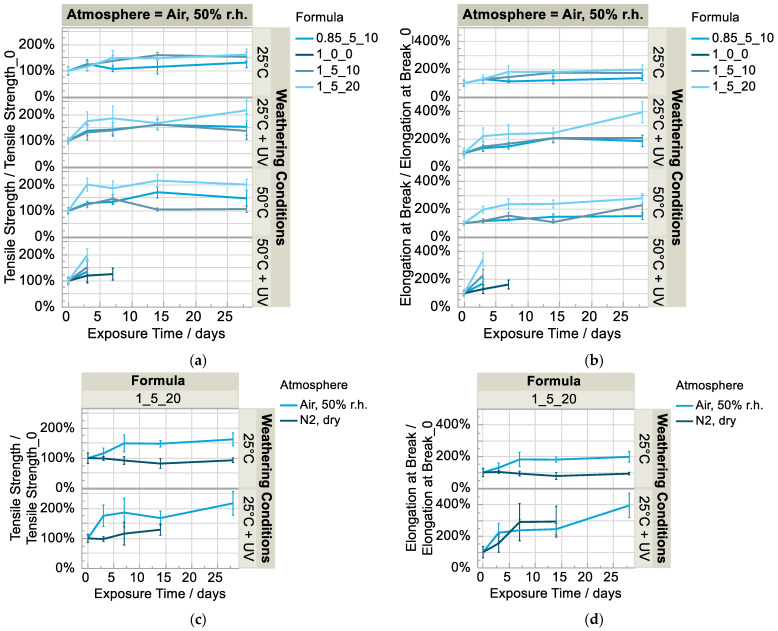
(**a**) Normalized tensile strength and (**b**) elongation at break in humid air over time and (**c**) normalized tensile strength and (**d**) normalized elongation at break in dry N_2_ atmosphere vs. humid air (n = 5).

**Figure 4:**
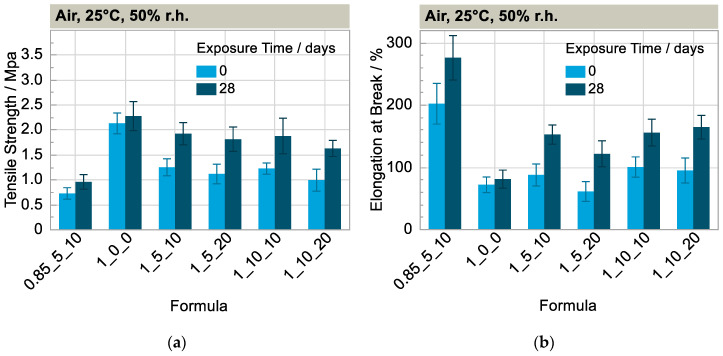
(**a**) Tensile Strength and (**b**) elongation at break after 0 and 28 days of weathering at 25 °C and 50% r.h. (n = 5).

Looking at the development of the tensile strength and elongation at break normalized to the initial value (Figure 3a,b), it is noticeable that both characteristic values of the materials with additives increase sharply at the beginning of the weathering phase compared to the reference without additives (1_0_0). The formula with a high solvent content (1_5_20) shows a particularly great increase compared the formulations with a low solvent content (1_5_10 and 0.85_5_10). Two explanatory approaches are discussed below. The first approach is that the solvent partially inhibits the crosslinking reaction, and the crosslinking progresses with the successive loss of solvent. This is supported by the fact that the tensile strength of all formulations increased strongly and approached that of the polyurethane matrix without additives (1_0_0) after 28 days of weathering at 25 °C and 50% r.h. (except formulation f (0.85_5_10)), depicted in Figure 4a. This is consistent with the observed tensile strength and elongation at break remaining approximately the same in the dry nitrogen atmosphere (25 °C, without irradiation) in which the solvent does not escape, as shown above (Figure 3c,d). For post-crosslinking, however, free isocyanate groups would still have to be present. In this case, intense bands would be visible in the infrared spectrum of the sample at approximately 2270 cm^−1^ [17]. This is not the case (Figure 2a). Furthermore, the elasticity should decrease with further curing, which is also not the case (Figure 7a). The second explanatory approach is that the free volume decreases with the loss of solvent and therefore the secondary valence forces of the matrix polymer chains increase, especially the urethane-NH-ether oxygen bonds. This assumption seems plausible with the observation that the properties change particularly strongly in the formulation with a high solvent content (1_5_20) compared to the initial state.

The specimens weathered at 50 °C and 50% irradiation intensity were so soft and sticky on the surface already after seven days (Figure 6) that they regularly slipped out of the restraint during the tensile test; no valid values resulted except for the reference specimens (Figure 3a,b). The same was observed for those specimens irradiated at lower temperature (25 °C) and in the dry nitrogen atmosphere (Figure 3c,d). Tensile testing after 28 days was no longer possible. However, the same specimens aged under air atmosphere apparently degraded more slowly and could be measured.

**Figure 5:**
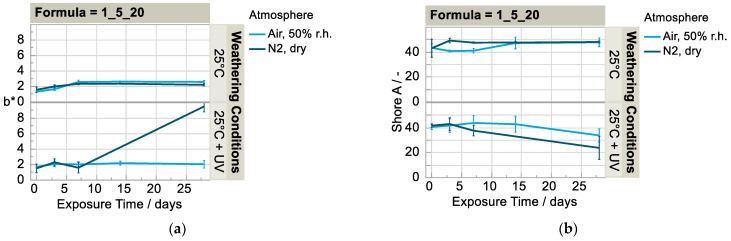

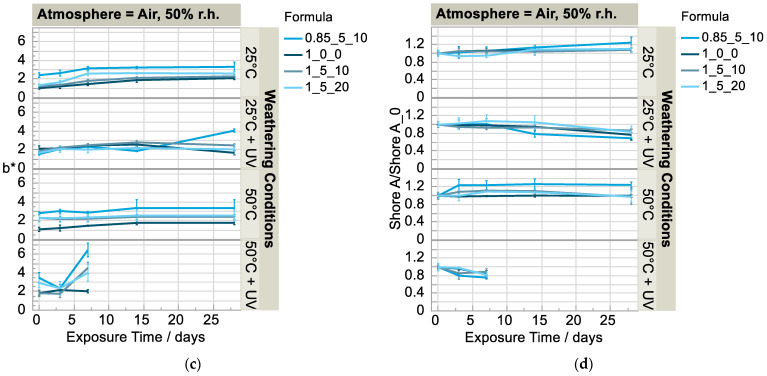
Development of (**a**) b* value (n = 3) and (**b**) Shore A value (n = 6) in humid air vs. dry nitrogen atmosphere at 25 °C with and without irradiation; development of (**c**) b* value (n = 3) and (**d**) normalized Shore A value (n = 6) in humid air over time.

The fact that the material in the case of irradiation degrades faster under the dry nitrogen atmosphere than under the humid air atmosphere is impressively shown through the comparison of the yellow value and the hardness (Figure 5a,b). While the initial value for b* is, on average, approximately 1.5 in both cases, it increases to approximately 9.5 under dry nitrogen atmosphere and increases only slightly to an average of 2.0 under the humid air atmosphere (Figure 5a). A slight increase in the yellowness value can also be observed during weathering without irradiation, both in the air and in nitrogen atmospheres (Figure 5a) and equally at different temperatures (25/50 °C) in the humid air (Figure 5c). This increase also seems to be independent of the formulation: the reference samples of the polyurethane matrix (1_0_0) increase to a similar extent, although at a somewhat lower level due to the lack of additives. The results of the weathering variant 25 °C with irradiation are not plausible in that the yellowness values of all formulations increase slightly within the first two weeks and decrease again to approximately the initial value in the following two weeks with the exception of formulation f (0.85_5_10). The reason for this could be a disturbing factor during the color measurement, which led to systematically lower values. For example, an incorrect calibration before the measurements or an exposure to extraneous light during the measurement. This is supported by the fact that the values of L* and a* (not shown here) were also systematically lower.

**Figure 6:**
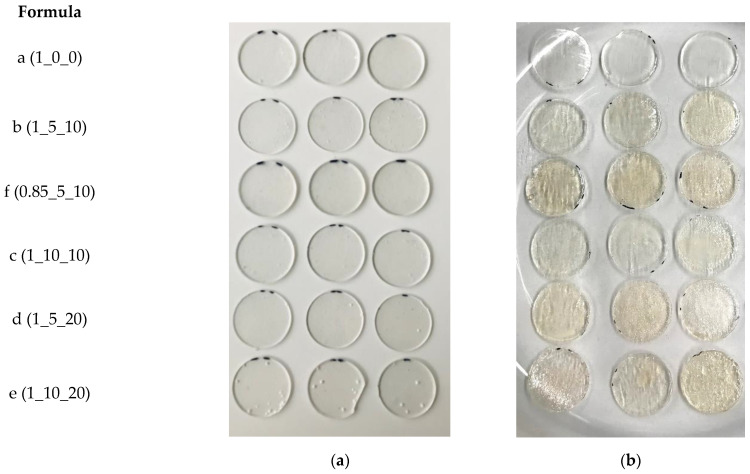
Photograph of degraded specimens (**a**) after four weeks of weathering at 50 °C/50% r.h. in the air and (**b**) after seven days of weathering at 50 °C/50% r.h and 50% irradiation intensity in air atmosphere.

**Figure A1:**
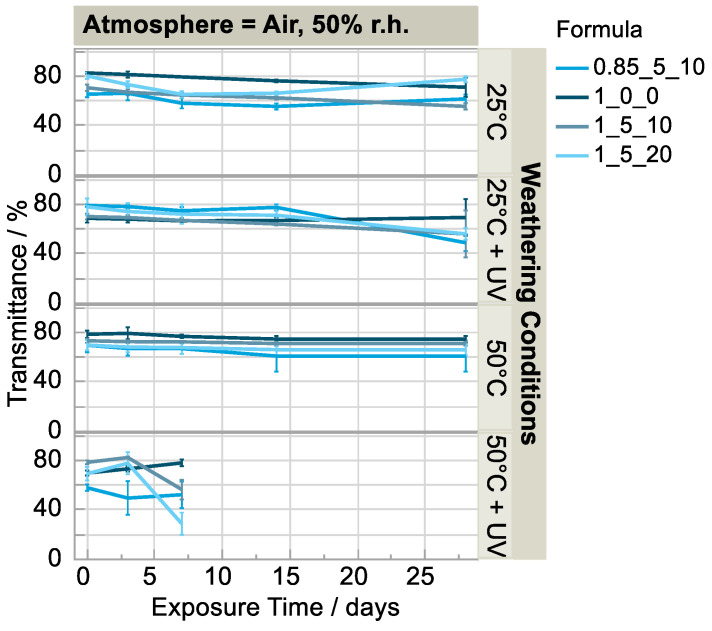
Visible transmittance values at weathering in humid air over time.

As expected, irradiation and elevated temperature have a synergetic effect on degradation. Thus, at 50 °C and irradiation as described, all formulations were already degraded after seven days to such an extent that tensile tests were no longer possible (c.f. Figure 6). This is particularly evident from the sharp increase in the yellow values (Figure 5c) and the drop in the Shore A hardness (Figure 5d) within the first seven days. Only in the reference formula a (1_0_0) did the yellowness value not change significantly during this period, while the hardness changed to the same extent as in the other samples with additives. The pure PUR matrix remains almost colorless, even at elevated temperature with irradiation, as the only formulation tested. This finding fits the literature because polyurethanes in which an aliphatic HDI is used are considered particularly lightfast [9,10]. Accordingly, radiation-induced yellowing seems to occur only in the presence of the additives. The same applies to the transmittance spectra (Figure A1, Appendix A). When weathered with irradiation, a strong decrease in transmittance is observed for the formulations with additives but not for the reference sample. At an elevated temperature (50 °C), this change occurs faster compared to the lower temperature (25 °C). The transmittance loss can be explained by the yellowing and the structural change of the surface (more reflection). 

The hypothesis derived from the literature was that the solvent intensifies the degradation processes [16]. However, the results show no influence of the initial solvent content on the degradation and yellowing; however, this may be due to the loss of propylene carbonate under the humid atmosphere shown above. In contrast, the result when weathered under a dry atmosphere in which the solvent remains almost completely supports the hypothesis since after four weeks at 25 °C, the b*-value increased to 9.5, while the b* value under humid air atmosphere remained unchanged at 2. This indicates that either the yellowing not necessarily results from oxidative processes or that sufficient oxygen was nevertheless available. This could have several reasons. On one hand, the wide-meshed cross-linked PUR matrix itself could have contained enough oxygen; on the other hand, the container could have been leaky, which is unlikely in view of the differences in solvent loss. However, the seals and the adhesion of the quartz glass could have allowed for oxygen diffusion. Furthermore, oxygen could have remained in a sufficient quantity in the container during the filling process despite concentration measurement. In addition, the container was regularly opened for sampling and the samples were briefly exposed to ambient air.

Formulation f (0.85_5_10) is also particularly striking as its yellow value increases more strongly and the hardness value decreases more strongly with irradiation than for all other formulations. This is the only formulation that yellows significantly, even at low temperatures of 25 °C and under irradiation, but only in the course of the last two weeks of the weathering period. The Shore A hardness also decreases particularly strongly to approx. 70% of the initial value. Additionally, during weathering without irradiation, the hardness values of this formulation increase comparatively strongly by more than 20% on average, whereby this increase at 50 °C is already reached after three days and at 25 °C only after four weeks. The increasing hardness values can be explained by the loss of solvent. Decreasing hardness values can be attributed to changes in the surface properties, which occur mainly with irradiation and more rapidly with an elevated temperature. As described in the introduction, a photo-initiated radical chain reaction may occur upon irradiation, with generally more degradation to be expected at the surface due to radiation absorption and oxygen availability.

**Figure 7:**
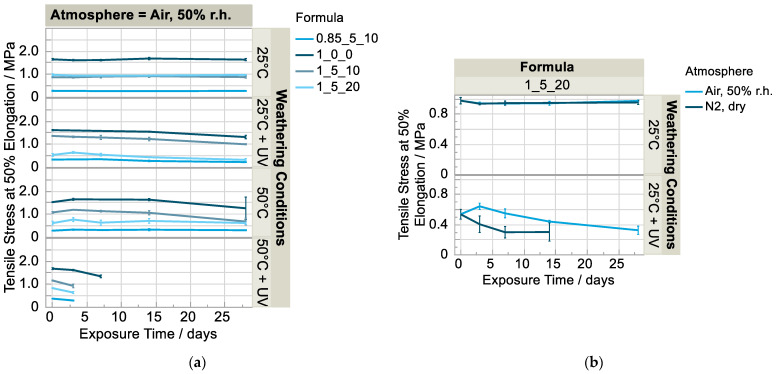
Change in elasticity over exposure time in (**a**) humid air and (**b**) humid air and dry nitrogen in comparison (n = 5).

The change in elasticity, here demonstrated by comparing the stress at 50% elongation, is used as a measure of how far the material as a whole was structurally degraded. For the formulation d (1_5_20), for example, after three days at 50 °C and under irradiation this value decreases significantly from an average of 0.84 MPa to 0.64 MPa, which is 24% less (Figure 7a). Irradiation at a lower temperature seems to lead to similar results, but the degradation process needs a longer induction time because significant changes are observed only after one or two weeks. In contrast, the elasticity does not change significantly after four weeks of weathering at 25 °C without irradiation in either humid air or in dry nitrogen (Figure 7a,b). When the specimens are irradiated in a dry nitrogen atmosphere, a significantly faster decrease in elasticity is observed, as is the case for hardness (Figure 7b). At 50 °C without irradiation, no clear correlation can be found. Thus, the radiation-induced chain scission does not appear to be just a surface effect but rather leads to a decrease in the crosslink density in the material and thus to a reduction in elasticity and an over-proportional increase in elongation at break. The sticky and viscous sample surface (Figure 6) is most likely composed of low molecular weight degradation products which are no longer bound to the cross-linked matrix. In order to detect structural changes, such as chain scission or oxidation products on the surface, the infrared spectra of the materials are evaluated.

**Figure 8:**
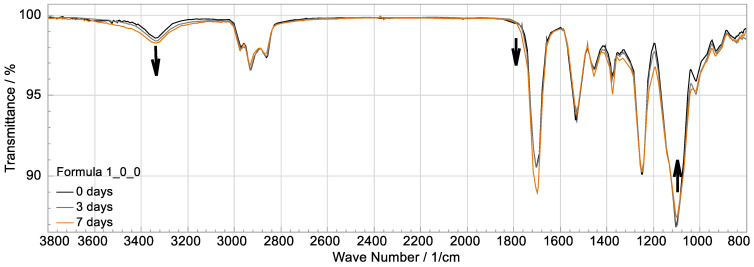
Overlapping FTIR curves of the reference material after different weathering durations at 50 °C, 50% r.h. and irradiation.

Figure 8 shows an example of the superimposed infrared spectra of the reference material during the harshest conditions of weathering at 50 °C and under irradiation in moist air at different times. The peaks at 1220, 1530 and 1695 cm^−1^ are characteristic of polyurethanes and are due to valence vibrations within the urethane group, e.g., the vibration of the CO double bond at 1695 cm^−1^ [17]. Except for the peak at 1530 cm^−1^, no particularly pronounced change can be seen, so it can be assumed that the urethane bonds are intact, which is consistent with the literature considering the aliphatic HDI as an isocyanate component. The peak at 1100 cm^−1^ can be attributed to the ether bond (-C-O-C-) in the polyol component [17]. This is less pronounced in the colored curve than in the other two samples. Thus, ether bonds are likely to have degraded in the material. According to literature, ether bonds have a high tendency to oxidize [12]; therefore, the oxidative degradation of the ether bonds can be assumed. If this is the case, any oxidation products should be detectable. Oxidation products with hydroxyl groups and hydroperoxides absorb at 3200–3700 cm^−1^ and carbonyl groups absorb at 1705–1790 cm^−1^ [10]. This is precisely where a correspondingly higher absorption is observed (Figure 8). This observation could also be made for weathering at 25 °C and under irradiation but only for longer exposure times, correlating with a decreasing hardness and elasticity. In the case of weathering without irradiation, no deviations occur in these areas. 

**Figure 9:**
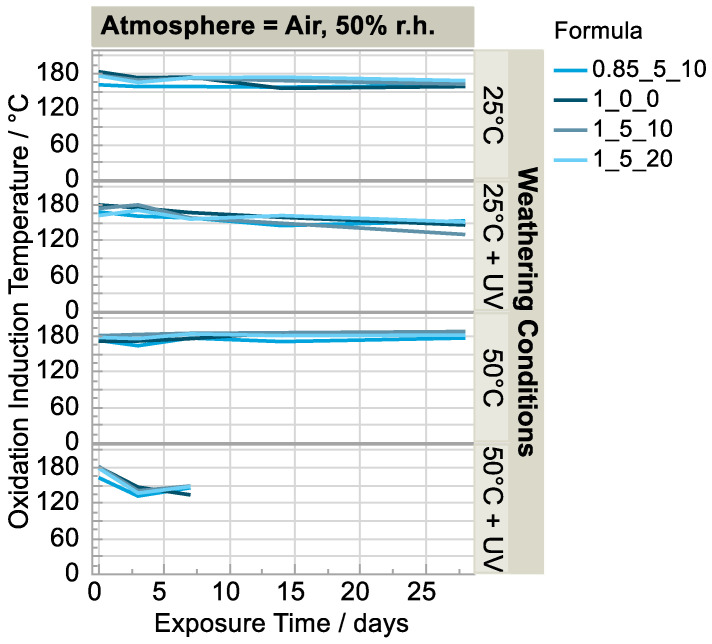
Development of OIT weathered in humid air atmosphere (n = 1).

The results of the dynamic OIT analysis support the hypothesis of a photo-initiated radical chain reaction that leads to the oxidative degradation (Figure 9). With an increasing exposure time to UV light, a steadily decreasing OIT can be seen, occurring faster at higher temperatures. A plausible explanation is that the longer the exposure duration, the more advanced the chain reaction and thus the concentration of hydroperoxides. This in turn leads to a decreasing OIT.

## 4. Conclusions

A significant effect of weathering on the electrolyte samples is the almost complete loss of the solvent propylene carbonate at standard climate after a few days, what was not expected to this extent. This loss does not happen under the dry nitrogen atmosphere, which is why it is assumed that the solvent migrates into the humid ambient air due to its good solubility in water. The loss leads to the rapid decrease of the ionic conductivity in which the electrolyte formulation with sub-stoichiometric matrix (0.85_5_10) turns out to be advantageous because it retains a comparatively high ionic conductivity. Furthermore, the loss leads to the increase in the strength and elongation at break, which is explained by the decrease in the free volume and the corresponding increase in the secondary valence forces of the matrix polymer chains, especially the urethane NH-ether oxygen bonds. As suspected, the urethane linkages of the aliphatic PUR are stable to UV radiation and hydrolysis. The essential degradation mechanism appears to be the photo-oxidative degradation of the ether bonds of the polyether polyol as hypothesized, including chain scission within the radical chain reaction. This leads to structural changes in the material. In addition to the stickiness and the decrease in the Shore hardness at the surface, elasticity, i.e., the stress absorbed at an elongation of 50%, also decreases, indicating that the radiation-induced chain scission is not merely a surface effect, but rather leads to a decrease in the crosslink density in the material. The sticky and viscous sample surface is most likely composed of low molecular weight degradation products which are no longer bound to the cross-linked matrix. The expected lightfastness does apply to the pure PUR matrix, which does not yellow. However, as soon as the salt and solvent are present, yellowing occurs. The observed degradation proceeds faster at an elevated temperature. When exposed to weathering without (UV) irradiation, no significant changes in properties appear (apart from those due to solvent loss), supporting the hypothesis that thermo-oxidation of the ether bond does not occur even at slightly elevated temperatures (50 °C, 50% r.h.). The hypothesis that inert atmospheres slow down the degradation mechanisms due to the lack of oxygen could not be tested conclusively because there was likely too much (residual) oxygen in the sample container. On the contrary, the samples degrade even faster when irradiated. Since the solvent does not escape from these samples due to the dry atmosphere, this finding supports the assumption that the solvent propylene carbonate intensifies the degradation. The amount of salt has no effect on degradation at the selected levels in this study (5/10 wt%). To ensure that the electrolyte functions permanently, the solvent should be replaced, e.g., by an ionic liquid. Protection from UV radiation is also essential since the matrix degrades by photooxidation, and the degradation occurs faster in higher temperatures. This could be achieved by using foils with a UV-protective layer or, in case of the intended plastic back-injection molding of EC films, by using a granulate with a UV stabilizer. Complete isolation from ambient air to prevent oxidation with oxygen appears challenging. The substoichiometric matrix is advantageous in terms of ionic conductivity even with a complete loss of solvent.

## Figures and Tables

**Table 1:** Investigated electrolyte formulations, adapted from Ref. [6].

Formula	Formula Abbreviation	Stoichiometric Ratio of Polyurethane Matrix (OH/NCO)	Parts by Weight of Salt (LiTFSi)/wt%	Parts by Weight of Solvent (Propylencarbonate)/wt%
a	1_0_0	1	0	0
b	1_5_10	1	5	10
c	1_10_10	1	10	10
d	1_5_20	1	5	20
e	1_10_20	1	10	20
f	0.85_5_10	0.85	5	10

**Table 2:** Weathering conditions; * Nitrogen (N_2_), quality 5.0.

Electrolyte	Atmosphere	Humidity/% r.h.	Temperature/°C	Irradiation Intensity/%	Weathering Duration/Days
a–f	air	50	25	0	3/7/14/28
a–f	air	50	25	50	
a–f	air	50	50	0	
a–f	air	50	50	50	
d	N_2_ *	~0 *	25	0	
d	N_2_ *	~0 *	25	50	

## Data Availability

Data is available from the corresponding author on reasonable request.
